# Data on near infrared polarization spectroscopy measurements to evaluate the potential of the Mueller matrix elements in characterization of turbid liquid samples

**DOI:** 10.1016/j.dib.2019.103756

**Published:** 2019-03-07

**Authors:** A. Ducanchez, D. Heran, R. Bendoula

**Affiliations:** Montpellier SupAgro, Irstea, Univ. Montpellier, UMR ITAP, 34060 Montpellier, France

## Abstract

In this article, a set of 50 turbid liquid samples with different levels of absorption and scattering properties were prepared and measured at various orientations of polarizers and analyzers to obtain the 16 elements of the complete Muller matrix. Partial Least Square (PLS) was used to build calibration models in order to assess the potential of polarization spectroscopy through the elements of Muller matrix to predict chemical and physical parameters.

**Table:** Specifications table [*Please fill in right-hand column of the table below.*]

Subject area	*Optical Diffuse Spectroscopy, Chemistry, Scattering media.*
More specific subject area	*Polarization Light Spectroscopy*
Type of data	*Table, Figure, .mat file*
How data was acquired	*Polarized Light System with PSG (polarization state generator) and PSA (polarization state analyzer) coupled with Visible and Near Infrared Spectrometer (Zeiss MSS 1)*
Data format	*Raw and analyzed data*
Experimental factors	*50 turbid liquid samples that contain different levels of absorption and scattering properties were measured in different polarization states in order to obtain the elements of Muller matrix*
Experimental features	*Polarization light Spectroscopy coupled with chemometric analysis was used to assess the potential of Muller matrix to predict and to discriminate the concentration in absorbers and scatterers of turbid liquid samples*
Data source location	*IRSTEA, Montpellier, France (43° 36′ 38.768″ N 3° 52′ 36.178″ E)*
Data accessibility	*Data is available with this publication*
Related research article	*W.S. Bickel and W.M. Bailey, “Stokes vectors, Mueller matrices, and polarized light scattering,” Am. J.Phys. 53, 468–478 (1985).*

**Table:** 

**Value of the data** • These data describe the complete Muller matrix in visible (VIS) and near-infrared (NIR) wavelength range for different model turbid media. • These data establish a link between physical and/or chemical properties of various turbid liquid samples and VIS/NIR spectra in diffuse reflectance. • VIS/NIR spectroscopy in polarized light and multivariate analysis are able to predict physical and chemical properties of turbid media. • The experimental data of turbid media coupled with VIS/NIR spectra can be used as reliable database for testing and analyzing different PLS methods as multi-block regression (PO-PLS, SO-PLS … etc.) [1].

## 1 Data

**Fig. 1:**
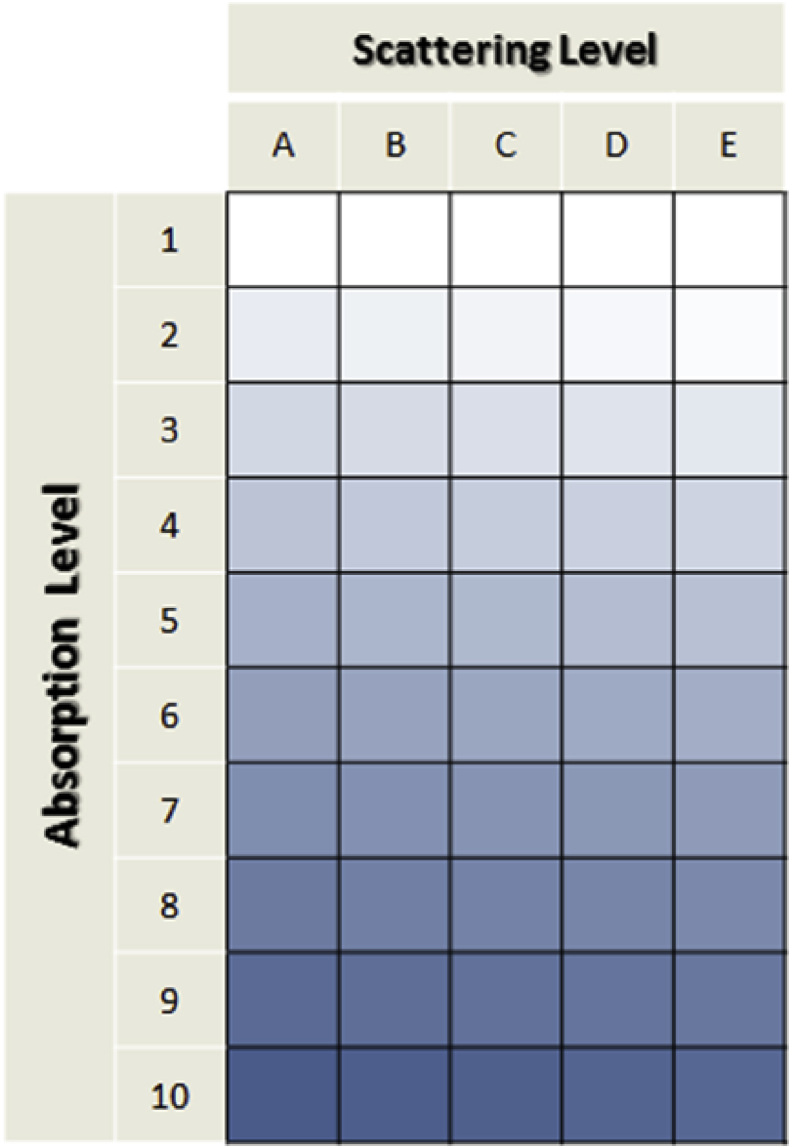
Set of 50 turbid liquid samples obtained by mixing MB (absorber), IL (scatterer) and distilled water (dilution agent). For rows, MB concentrations increases from top to bottom (0, 4, 8, 16, 20, 32, 48, 64, 80 and 128 μM) and for columns, IL concentrations increases from left to right (0.227, 0.454, 0.908, 1.816 and 3.682%).

**Fig. 2:**
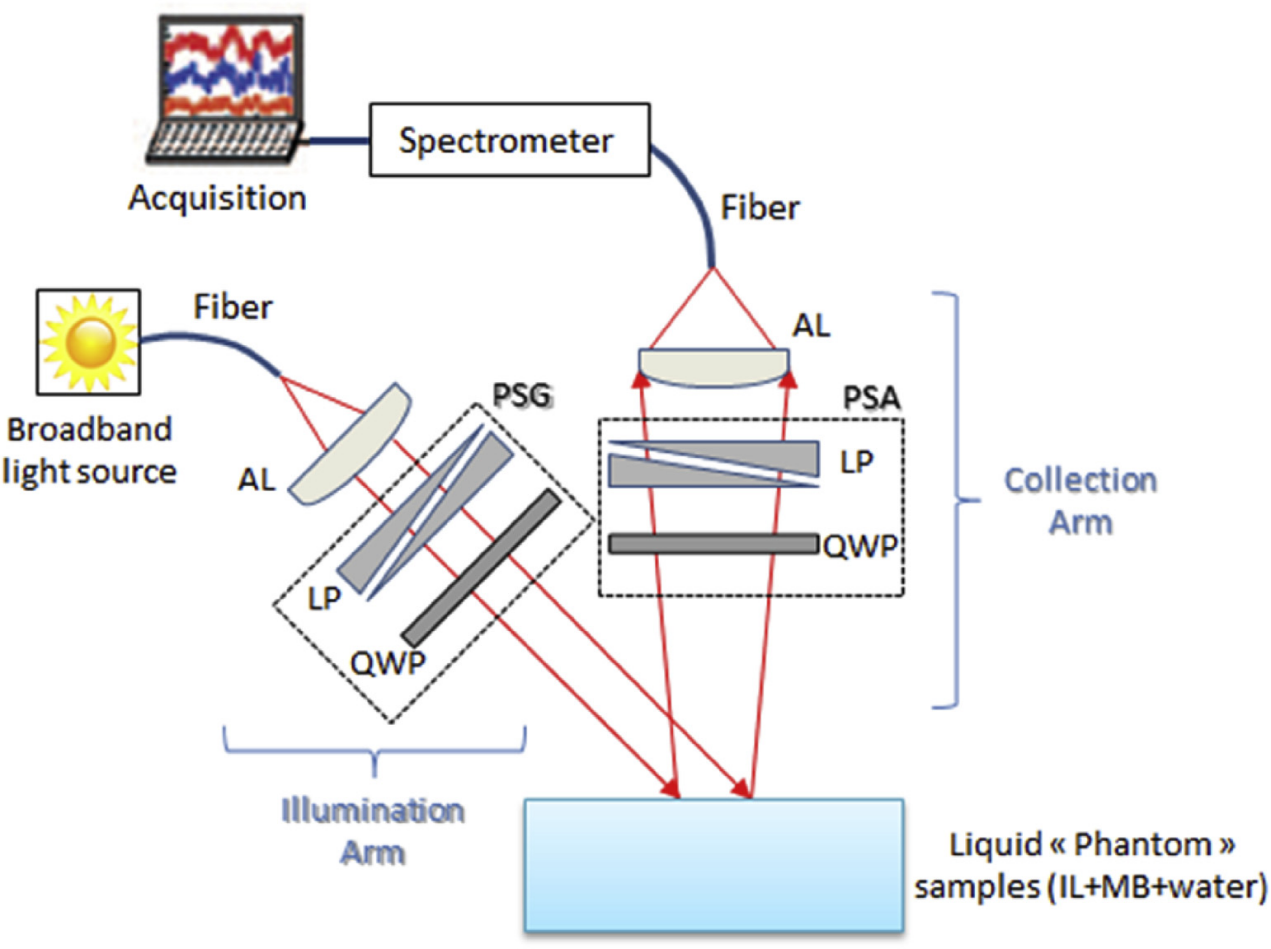
Schematic representation of Polarized Light Spectroscopy (PoLiS) system in reflexion [4] (PSG = polarization state generator, PSA = polarization state analyzer, LP = linear polarizer, QWP = quarter wave plate and AL = aspheric lens).

**Fig. 3:**
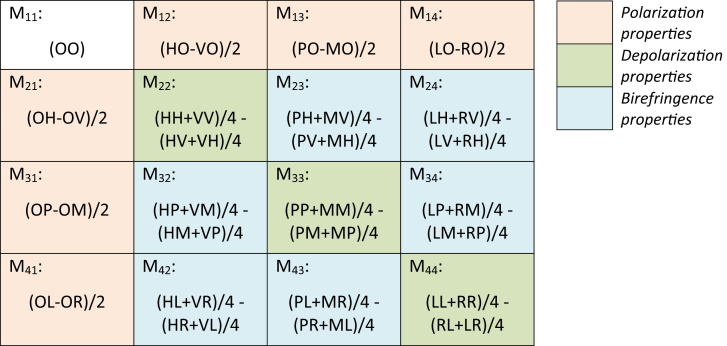
This table details how to obtain the different elements of the Muller matrix from combinations of different states of polarization [5]. A two letter combination corresponds to one measurement. For example, the combination (VP) means that the PSG is adjusted to obtain linear polarization along the vertical axis (y axis) for incoming light and PSA is adjusted to recover linear polarization with a + 45° offset for reflected light.

**Fig. 4:**
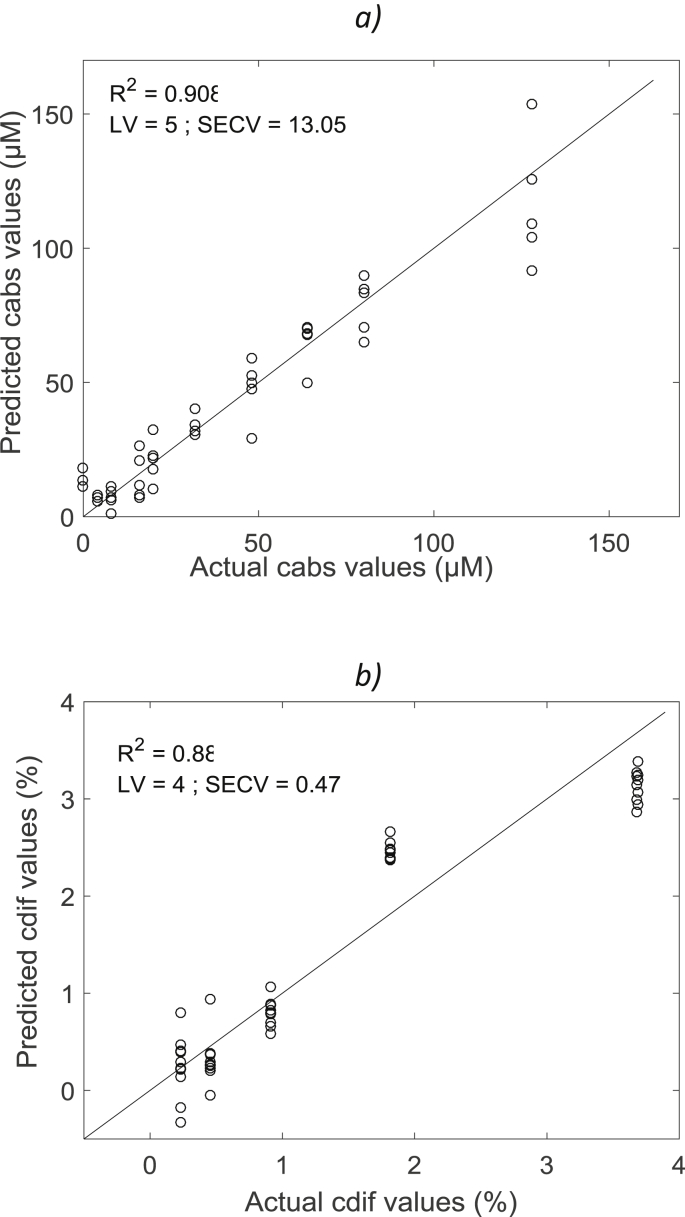
Calibration model for the concentrations of methylen blue absorbers (c_abs_) in μM (a) and the concentrations of intralipid scatterers (c_dif_) in % (b) of element M_22_ of the Muller matrix.

Several measurements on 50 turbid liquid samples with different level of concentration in scatterers and absorbers were made (Fig. 1). In parallel, diffuse reflectance spectra in different states of polarized light in visible and near-infrared wavelength range were measured with Polarized Light System (Fig. 2). From these spectral raw data, linear combination of these different polarization states were calculated to obtain the complete Mueller matrices (Fig. 3) for each 50 turbid liquid samples corresponding to the processed data set. The details of all databases are given in part 3. With a Partial Least Square (PLS) algorithm, an example of predicting models were obtained for the concentration of absorbers (Fig. 4a) and scatterers (Fig. 4b) for element M_22_ of the Muller matrix.

## 2 Experimental design, materials, and methods

### 2.1 Liquid phantom

Turbid liquid samples were composed of methylene blue (MB), Intralipid 20% solution (IL) and distilled water used as absorbing, scattering and dilution material respectively [2]. IL is an intravenous fat emulsion that contains fat globules which act as scattering particles. MB is a water-soluble non-scattering dye that presents two peaks absorption at 668 nm and 609 nm due to monomer and dimmer forms in aqueous solutions [3]. In contrast, the absorption by water and IL is minimal in the wavelength range. Hence, MB and IL are well-adapted in the considered wavelength range to interpret and to discriminate the effects of scattering and absorption on the measured polarized reflectance spectra.

### 2.2 Samples preparation

A set of 50 turbid liquid samples were prepared by mixing in different ratios 10 concentration of the absorber (0, 1, 2, 4, 5, 8, 12, 16, 20 and 32 mL of a 400 μM stock solution) relating to row number with 5 concentrations of the scattered (1, 2, 4, 8 and 16 mL of the IL 20% solution) relating to column letter [Fig. 1.]. According to this experience plan, the liquid samples have MB concentration of 0, 4, 8, 16, 20, 32, 48, 64, 80 and 128 μM respectively with increasing row number and a scattering particles concentration of 0.227, 0.454, 0.908, 1.816 and 3.682% (*v/v*) respectively with increasing column letter. Each sample was conditioned in a beaker and distilled water was added to obtain 100 mL for all liquid samples. At last, each sample was rigorously mixed to homogenize the aqueous solutions before the spectral measurements.

### 2.3 Polarization spectroscopy system

The experimental setup for studying the diffuse reflectance of polarized light is presented in Fig. 2. This system was the same Polarized Ligth Spectroscopy system (PoLiS) that we used in previous study [4]. The PoLiS system integrates a polarization state generator (PSG) and polarization state analyzer (PSA). Both the PSG and PSA consisted of a rotating broad-band (400–800 nm) linear polarizer (LP) (NT52-557, Edmunds Optics) and a rotating quarter-wave plate (QWP) (AQWP05M − 600, Thorlabs) in order to generate and to select the various polarization states that are needed. Spectral data were collected with a spectrometer (MMS1, Zeiss) in the 350–1100 nm wavelength range at 3 nm intervals.

### 2.4 Mueller matrix

The Mueller matrix provides the most general and complete description of the response of a medium to excitation by polarized light in either reflection or transmission configurations. Fig. 3 lists the necessary measurements and combinations in order to determinate each matrix element [5]. To obtain the 16 elements of the complete Mueller matrix, it was necessary to measure 7 configurations respectively for both the PSG and PSA:

• O: unpolarized
• H: linearly polarized along the horizontal axis (x axis),
• V: linearly polarized along the vertical axis (y axis),
• P: linearly polarized with a +45° offset,
• M: linearly polarized with a −45° offset,
• L: left-handed circularly polarized,
• R: right-handed circularly polarized,

From these 49 configurations in total corresponding to 49 reflectance spectral measurements (7 for incoming polarized light x 7 for reflected polarized light), each matrix element was calculated by the corresponding linear combinations. For example, the M_12_ element is obtained by measuring the total reflected intensity for an incident light linearly polarized along the horizontal axis and subtracting from this the total reflected intensity for an incident light linearly polarized along the vertical axis. Finally, except for the first element, each other element of the Muller matrix corresponding to physical properties of the medium considered such as polarization, depolarization or birefringence.

### 2.5 Spectral acquisition

For each turbid liquid samples, diffuse reflectance spectra of polarized light was measured for each of the 49 configurations from the both PSG and PSA. Moreover, for each of these 49 configurations, white diffuse standard (Spectralon®, SRS-99-010. Labsphere) was measured in order to standardize spectra from non-uniformities of all components of the PoLiS system.

### 2.6 PLS algorithm

All computations and multivariate data analysis were performed with Matlab software v. R2015b (The Mathworks Inc., Natick, MA, USA). A Partial Least Square (PLS) algorithm was used to model the physical and chemical parameters of the turbid liquid media. A general PLS model was built using the whole calibration set (two third of the sample) and a predicting set (one third of the sample). The number of Latent Variables (LV) was determined by comparing performances by leave-one-out cross validation method. Two basic statistical parameters including the determination coefficient (R^2^) and the standard error of cross-validation (SECV) were calculated. These parameters were used to assess the performance of each calibration model for predicting absorbers and scatterers concentration. An example of predicting models for element M_22_ of the Muller matrix is presented for the concentration of absorbers (Fig. 4a) and scatterers (Fig. 4b).

## 3 Details on the data available

In this publication, several database matrices in matlab file are available:

- “raw_spectral_data”: a zip file that contains 50 matlab files corresponding to the 50 turbid liquid samples. Each matlab file contains 49 raw spectral data in diffuse reflectance from the 49 possible configurations (OO, OR, …, VV.) through the different polarization states at the input and output of the PoLiS system necessary to create the complete Mueller matrix.

- “cabs”: matrix with 50 rows and 1 column corresponding to the concentrations of absorbers in μM for the 50 turbid liquid samples with this structuring:

$$cabs=\left[\begin{array}{c} \begin{array}{c} \begin{array}{c} \begin{array}{c} A1\\ B1\\ C1 \end{array}\\ D1\\ E1 \end{array}\\ A2\\ B2 \end{array}\\ \vdots \\ E10 \end{array}\right]=\left[\begin{array}{c} \begin{array}{c} \begin{array}{c} \begin{array}{c} 0\\ 0\\ 0 \end{array}\\ 0\\ 0 \end{array}\\ 4\\ 4 \end{array}\\ \vdots \\ 128 \end{array}\right]$$

Note that the concentration of absorbers increases with the number.

- “cdif”: matrix with 50 rows and 1 column corresponding to the concentrations of scatterers in % for the 50 turbid liquid samples with this structuring:

$$cdif=\left[\begin{array}{c} \begin{array}{c} \begin{array}{c} \begin{array}{c} A1\\ B1\\ C1 \end{array}\\ D1\\ E1 \end{array}\\ A2\\ B2 \end{array}\\ \vdots \\ E10 \end{array}\right]=\left[\begin{array}{c} \begin{array}{c} \begin{array}{c} \begin{array}{c} 0.227\\ 0.454\\ 0.908 \end{array}\\ 1.816\\ 3.682 \end{array}\\ 0.227\\ 0.454 \end{array}\\ \vdots \\ 3.682 \end{array}\right]$$

Note that the concentration of scatterers increases with the letter.

- “m11, m22 … m14”: 16 matrices corresponding to the 16 elements (4 × 4) of the Mueller matrix for each of the 50 liquid phantom samples. Each matrices consists of 50 rows (50 liquid phantom samples) and 256 columns (256 wavelength of spectrometer in the 303–1146 nm wavelength range at 3.3 nm intervals).

For example, m11 matrix integrates the first element of each of Muller's 50 matrices and is therefore structured as follows:

$$m11=\left[\begin{array}{c} \begin{array}{c} \left[A1;\lambda_{303}\right]\\ \left[B1;\lambda_{303}\right]\\ \left[C1;\lambda_{303}\right] \end{array}\\ \left[D1;\lambda_{303}\right]\\ \begin{array}{c} \left[E1;\lambda_{303}\right]\\ \left[A2;\lambda_{303}\right]\\ \begin{array}{c} \left[B2;\lambda_{303}\right]\\ \vdots \\ \left[E10;\lambda_{303}\right] \end{array} \end{array} \end{array}\begin{array}{c} \begin{array}{c} \left[A1;\lambda_{306}\right]\\ \left[B1;\lambda_{306}\right]\\ \left[C1;\lambda_{306}\right] \end{array}\\ \left[D1;\lambda_{306}\right]\\ \begin{array}{c} \left[E1;\lambda_{306}\right]\\ \left[A2;\lambda_{306}\right]\\ \begin{array}{c} \left[B2;\lambda_{306}\right]\\ \vdots \\ \left[E10;\lambda_{306}\right] \end{array} \end{array} \end{array}\begin{array}{c} \begin{array}{c} \ldots \\ \ldots \\ \ldots \end{array}\\ \ldots \\ \begin{array}{c} \ldots \\ \ldots \\ \begin{array}{c} \ldots \\ \ldots \\ \ldots \end{array} \end{array} \end{array}\begin{array}{c} \begin{array}{c} \left[A1;\lambda_{1146}\right]\\ \left[B1;\lambda_{1146}\right]\\ \left[C1;\lambda_{1146}\right] \end{array}\\ \left[D1;\lambda_{1146}\right]\\ \begin{array}{c} \left[E1;\lambda_{1146}\right]\\ \left[A2;\lambda_{1146}\right]\\ \begin{array}{c} \left[B2;\lambda_{1146}\right]\\ \vdots \\ \left[E10;\lambda_{1146}\right] \end{array} \end{array} \end{array}\right]$$

- “r11, r22 … r14”: 16 matrices corresponding to “m11, m22 … m14” matrices where each element was divided by the corresponding spectral reference in order to normalize the spectral measurements.

- “lbd”: the 256 wavelengths of spectrometer.
