# Supplementary material for: Data on near infrared polarization spectroscopy measurements to evaluate the potential of the Mueller matrix elements in characterization of turbid liquid samples
Source: Data Brief. 2019 Mar 7;23:103756. doi: 10.1016/j.dib.2019.103756 (PMC6660517; doi:10.1016/j.dib.2019.103756)
Supplement: Supplementary file 1 — Multimedia component 1 [file mmc1.docx]

Conflict of Interest and Authorship Conformation Form

Manuscript title: “Data on near infrared polarization spectroscopy measurements to evaluate the potential of the Muller matrix elements in characterization of turbid liquid samples”

Please check the following as appropriate:

- All authors have participated in (a) conception and design, or analysis and interpretation of the data; (b) drafting the article or revising it critically for important intellectual content; and (c) approval of the final version.
- This manuscript has not been submitted to, nor is under review at, another journal or other publishing venue.
- The authors have no affiliation with any organization with a direct or indirect financial interest in the subject matter discussed in the manuscript
- The following authors have affiliations with organizations with direct or indirect financial interest in the subject matter discussed in the manuscript:

Author’s name Affiliation

Arnaud DUCANCHEZ Montpellier SupAgro, Irstea - UMR ITAP

Ryad BENDOULA Irstea - UMR ITAP

Daphné HERAN Irstea - UMR ITAP


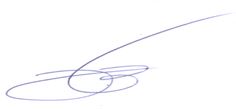


Signature Date

10-01-2019
